# Survival Analysis in Endometrial Carcinomas by Type of Surgical Approach: A Matched-Pair Study

**DOI:** 10.3390/cancers14041081

**Published:** 2022-02-21

**Authors:** Pluvio J. Coronado, Agnieszka Rychlik, Laura Baquedano, Virginia García-Pineda, Maria A. Martínez-Maestre, Denis Querleu, Ignacio Zapardiel

**Affiliations:** 1 Women’s Health Institute, Hospital Clínico San Carlos, IdISSC, Complutense University, 28040 Madrid, Spain; plujcoro@ucm.es; 2 Gynecologic Oncology Unit, Maria Sklodowska-Curie National Research Institute of Oncology, 00-001 Warsaw, Poland; 3 Department of Obstetrics and Gynecology, Miguel Servet Hospital, 50009 Zaragoza, Spain; lbaquedanome@hotmail.com; 4 Gynecologic Oncology Unit, La Paz University Hospital—IdiPAZ, 28046 Madrid, Spain; virginia.garciapineda@gmail.com (V.G.-P.); ignaciozapardiel@hotmail.com (I.Z.); 5 Department of Obstetrics and Gynecology, Hospital Virgen del Rocío, 41013 Sevilla, Spain; martinez.maestre@hotmail.com; 6 Division of gynecologic surgery, Hopital de Hautepierre, 67200 Strasbourg, France; denis.querleu@esgo.org; 7 Department of Obstetrics and Gynecology, Policlinico Agostino Gemelli, 00168 Rome, Italy

**Keywords:** endometrial carcinoma, survival, robotic-assisted laparoscopy, laparoscopy, laparotomy, morbidity, open approach

## Abstract

**Simple Summary:**

We carried out a retrospective cohort study of 1382 women diagnosed with endometrial cancer (EC). A total of 684 (49.5%) were operated on by minimally invasive surgery (MIS), 233 (34%) by robotic-assisted laparoscopy (RAL), 451 (66%) by conventional laparoscopy (LPS), and 698 (50.5%) by open surgery (OP). Disease-free (DFS), overall (OS), and specific survival related to EC (SS) outcomes were significantly higher for MIS compared to OP. When matched by age, BMI, co-morbidities, ASA score, histological type, grade, myometrial invasion, and FIGO stage, the DFS, OS, and SS amounts were similar between the MIS and OP groups. The surgical approach for women with EC does not impact disease-free or overall survival amounts when matched by homogeneous groups, but laparoscopy seems to offer a higher specific overall survival rate compared to the open surgery approach.

**Abstract:**

(1) Background: This study aimed to analyze the impact of surgical approach on survival rates in women diagnosed with endometrial cancer. (2) Methods: A retrospective multicenter cohort of 1382 women diagnosed with EC was performed. A total of 684 (49.5%) women underwent minimally invasive surgery, 233 (34%) underwent robotic-assisted laparoscopy (RAL), 451 (66%) underwent conventional laparoscopy (LPS), and 698 (50.5%) underwent open surgery (OP). Sociodemographic features, tumor characteristics, and survival rates were analyzed in the whole sample and in a matched-pair model. (3) Results: Women operated on by OP were significantly older, presented more comorbidities, and had more aggressive tumors. Disease-free (DFS), overall (OS), and specific survival related to EC (SS) amounts were significantly higher for MIS compared to OP (*p* < 0.001). When matched by age, body mass index, comorbidities, ASA score, histological type, grade, myometrial invasion, and FIGO stage, 798 patients were selected. DFS, OS, and SS amounts were similar between the MIS and OP groups. (4) Conclusions: The surgical approach for women with EC does not impact DFS or OS amounts when matched by homogeneous groups.

## 1. Introduction

Endometrial cancer (EC) is the most common gynecological neoplasm in developed countries. More than 75% of patients with EC are diagnosed at an early stage, with a 5-year overall survival rate of around 80% [1]. Recent studies in early-stage cervical carcinoma, (the phase III LACC trial and a large American epidemiological study) [2,3] have demonstrated an increased risk of recurrence and death in women who underwent minimally invasive surgery (MIS) in comparison to the open surgery approach (OP). These findings changed the gold standard of the surgical treatment of cervical cancer and suggest reconsidering whether MIS (robotic or laparoscopic) is safe in the management of other gynecological tumors. For EC, the Gynecologic Oncology Group (GOG) LAP2 study, a randomized trial that compared laparoscopy with laparotomy, did not show differences in oncological results between the two approaches [4]. Based on these results, standard laparoscopy (LPS) and robotic-assisted laparoscopy (RAL) have been adopted as the gold standard in endometrial cancer surgery. A recent Scandinavian population-based prospective cohort study showed that the incorporation of MIS reduced perioperative complications and improved survival when compared to open surgery [5].

Considering the new available evidence and the uncertainty of MIS in gynecological cancers, the aim of our study was to evaluate the impact of surgical approach (MIS or OP) on disease-free survival in women diagnosed with EC as a primary objective. As a secondary objective, we analyzed the impact on overall survival stratified by the type of MIS approach (robotic or laparoscopic).

## 2. Materials and Methods

### 2.1. Study Design and Participants

We conducted a multicenter retrospective cohort study of women diagnosed with preoperative apparent early-stage EC between 2005 and 2018 at four tertiary Spanish medical centers. LPS management of EC was initiated in 2005 in the majority of centers and RAL in 2007. The STROBE guidelines were followed to conduct this study [6].

We included all cases of apparent preoperative early FIGO stage EC that were managed at least with hysterectomy and bilateral salpingo-oophorectomy as primary treatment. Pelvic or pelvic and para-aortic lymph node dissections (LNDs) were performed based on current guidelines and surgical team experience [7,8]. We excluded cases with uterine sarcoma, synchronic ovarian cancer, and patients who were treated with radio- or chemotherapy as primary treatment.

In all cases, the same oncological team in each center performed all surgical interventions. The surgical approach depended on surgical team experience and the availability of robotic platforms in the centers. The LPS approach was performed in all centers, and RAL using a da Vinci Surgical System (Intuitive Surgical, Sunnyvale, CA, USA) was available only in two centers. The minimally invasive surgical approach in these two centers (robotic or laparoscopic) was selected depending on robot availability (usually once a week). The open surgery approach was indicated by surgical teams depending on vaginal size, uterus size, presence of adherences, and the patient’s tolerance to pneumoperitoneum, together with surgical team experience. Minimally invasive surgeries converted to laparotomies were included in the MIS group.

Preoperatively, mechanical bowel preparation was carried out at the surgical teams’ discretion, and prophylactic antibiotics and low molecular weight heparin were always administered. Intravenous fluids were maintained until patients tolerated oral fluids, usually within the first 24 h after the surgery. A Foley catheter was usually removed the day after the surgery. Patients were discharged if they demonstrated the ability to ambulate independently, tolerated a regular diet, had stable vital signs, and used oral analgesics for adequate pain control. The surgical pieces were analyzed by two expert pathologists in gynecological tumors from each center.

Adjuvant treatment was administered according to the European Society of Gynecologic Oncology guidelines and the Spanish Society of Gynecology and Obstetrics guidelines at moment of diagnosis [7,8]. External beam radiotherapy (EBRT), brachytherapy, and chemotherapy were indicated postoperatively after the agreement of each Institutional Tumor Board. The presence of recurrence and the patient status at last visit were recorded during the follow-up visits performed at least every 6 months.

### 2.2. Matched-Pair Model

**Figure 1:**
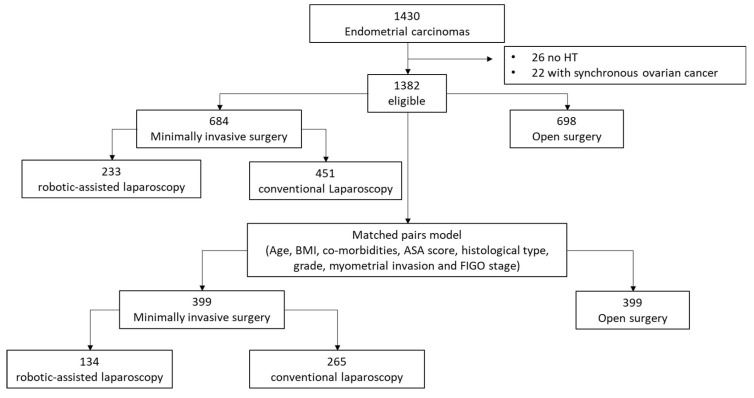
A flowchart indicating the selection of matched pairs.

To avoid the heterogenicity of a retrospective study, we performed a statistical model using matched pairs (1:1), which is the best approximation of a clinical trial in this type of study and avoids the possible bias in patient selection. The case-control match was designed to pair cases according to selected variables in order to homogenize both study groups (MIS vs. OP). In the election of the model, we selected variables that could have an impact on the choice of surgical approach, the use of adjuvant treatment, or patient survival. These variables were independent prognostic factors for disease-free survival (DFS) and overall survival (OS) in a Cox multivariate analysis (Supplemental Material Table S1), and were age at diagnosis, body mass index (BMI), pre-surgical comorbidities (including tobacco consumption, diabetes, cardiovascular diseases, thromboembolic events, chronic pneumopathies, and liver diseases), American Society of Anesthesiologists (ASA) score, histological subtype, histological grade, myometrial invasion, and FIGO stage. The flow chart indicating the selection of matched pairs and reasons for withdrawal is shown in Figure 1. A total of 798 patients (399 matched pairs) were included in the final analysis.

### 2.3. Statistical Analysis

A stratified analysis was performed comparing MIS vs. OP, as well as LPS vs. RAL vs. OP. Continuous variables were reported by mean and standard deviation, and comparisons between groups used Student’s t-test with normal distribution. Mann–Whitney tests were used for non-parametrical variables. Discrete variables were represented with absolute frequencies and relative percentages, and they were compared by a chi-squared test or Fisher’s exact test when appropriate.

For survival analysis, Cox’s method was used to assess the relation between the study groups and DFS, OS, or tumor-specific survival (SS). Multivariate analysis using Cox’s proportional hazards model was used to adjust the hazard ratio (HR) with a 95% confident interval (CI). Kaplan-Meier curves were used to estimate the survival distribution in the study groups. A log-rank test was used to calculate the statistical signification between the curves in relation to recurrence and death. All statistical tests were two-sided, and alpha error was set at 5%. All computations were performed using Stata v.11.2 (StataCorp LP, College Station, TX, USA) and IBM SPSS Statistics 25 (IBM Corp., Armonk, NY, USA).

## 3. Results

### 3.1. Whole Sample: MIS vs. Open Surgery

Out of 1430 women analyzed with primary early-stage EC diagnosed in the participant Spanish centers, a total of 1382 women who underwent surgical staging as primary treatment were included (Figure 1).

MIS (LPS or RAL) was performed in 684 (49.5%) cases. A total of 698 (50.5%) patients were operated on with open surgery. RAL was used in 233 women (16.6%) and LPS in 451 (36.6%). The general features of both study groups are shown in Table 1. In comparison to MIS, women who underwent open surgery were older (*p* = 0.003), had higher BMIs (*p* < 0.001), more comorbidities (*p* = 0.028), and worse ASA scores (*p* < 0.001). In relation to histopathological features, the tumors operated on by laparotomy were more frequently non-endometrioid (*p* < 0.001) and had a higher grade (*p* < 0.001), a higher rate of deep myometrial invasion (*p* < 0.001), lymphovascular space invasion (LVSI) (*p* = 0.002), and advanced FIGO stage (*p* < 0.001). When the cases were stratified by the European risk classification system [8], the cases operated on with an open surgery approach showed higher rates of high-risk and advanced metastatic tumors (*p* < 0.001). Adjuvant chemo- or chemo-radiotherapy were more frequently indicated in this study group (*p* < 0.001).

After a mean ± SD follow-up of 60.1 ± 40.5 months, 276 patients (20%) recurred, and 249 (18.0%) women died. A total of 172 (12.4%) deaths were related to EC, 14 (1.0%) deaths were caused by other intercurrent tumors, and 63 (4.6%) were due to other causes. Four patients were lost to follow-up. The follow-up was longer in the open surgery approach group than in the MIS group (*p* = 0.012). 

**Figure 2:**
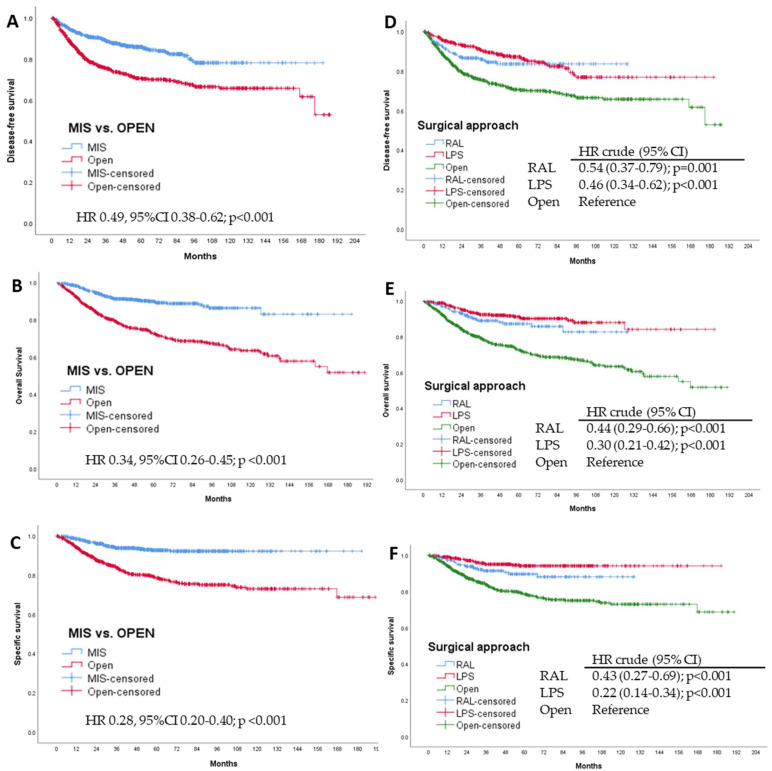
Kaplan-Meier curves for the surgical groups. The hazard ratio, 95% confidence interval, and corresponding *p*-values were estimated with the use of Cox’s proportional hazards model. MIS: minimally invasive surgery, indicating a laparoscopic (LPS) or robotic-assisted approach (RAL). Open: open surgery or laparotomic approach. (**A**,**D**) Disease-free survival curves, (**B**,**E**) overall survival curves, and (**C**,**F**) specific survival curves.

Kaplan-Meier curves showed a 5-year DFS rate of 85.9% in the MIS group, which was significantly higher than in the OP group (70.3%; HR 0.49, 95%CI 0.38–0.62; *p* < 0.001). The 5-year OS rate was 89.8% in the MIS group and 72.5% in the open surgery group (HR 0.34, 95%CI 0.26–0.45; *p* < 0.001). The 5-year specific survival percentage related to EC was 92.7% for MIS and 78.5% for the open surgery group (HR 0.28, 95%CI 0.20–0.40; *p* < 0.001) (Figure 2).

### 3.2. Whole Sample: MIS Subanalysis

When we analyzed the 684 women who underwent MIS by type of MIS (robotic vs. conventional LPS), we found that women who underwent RAL had worse ASA scores (*p* = 0.016), lower rates of LVSI (*p* = 0.039), lower para-aortic lymph node dissection (LND) rates (*p* < 0.001), lower rates of ESGO intermediate-risk tumors (*p* < 0.001), and received less adjuvant treatment (*p* = 0.012) (Table 2). 

In the survival analysis, follow-up was longer in the LPS group than in the RAL group (*p* = 0.013). Kaplan-Meier curves showed a 5-year DFS rate of 83.6% in the RAL group and 87.2% in the LPS group. The 5-year OS rate was 87.2% in the RAL group and 91.1% in LPS group. The 5-year survival related to EC rate was 89.6% for RAL and 94.1% in the LPS group. Survival was longer in the RAL and LPS groups in comparison to the open surgery group (Figure 2).

### 3.3. Matched-Pair Analysis: MIS vs. Open Surgery

A total of 399 matched pairs (798 women) of patients with endometrial cancer were included in the study. The MIS group counted for 134 patients managed by RAL and 265 patients managed by LPS. 

Patient characteristics, preoperative findings, pathologies, and therapeutic details are shown in Table 3. Both surgical groups were similar in all variables, except in follow-up, which was longer in the open surgery group (*p* = 0.012). When stratified by type of MIS, patients who underwent robotic surgery had higher histological grades (*p* = 0.028), lower rates of para-aortic LDN (*p* = 0.036), lower rates of ESGO intermediate-risk tumors (*p* = 0.001), and higher rates of chemo-radiation (*p* = 0.003).

After a mean ± SD follow-up of 58.7 ± 38.9 months, 55 (15.4%) women presented a relapse, and 94 (11.8%) patients died. Among the deceased patients, 62 cases (7.8%) were disease-related deaths, 28 (3.5%) were deaths because of other diseases, 4 (0.5%) were due to other intercurrent tumors, and 1 was lost to follow-up.

**Figure 3:**
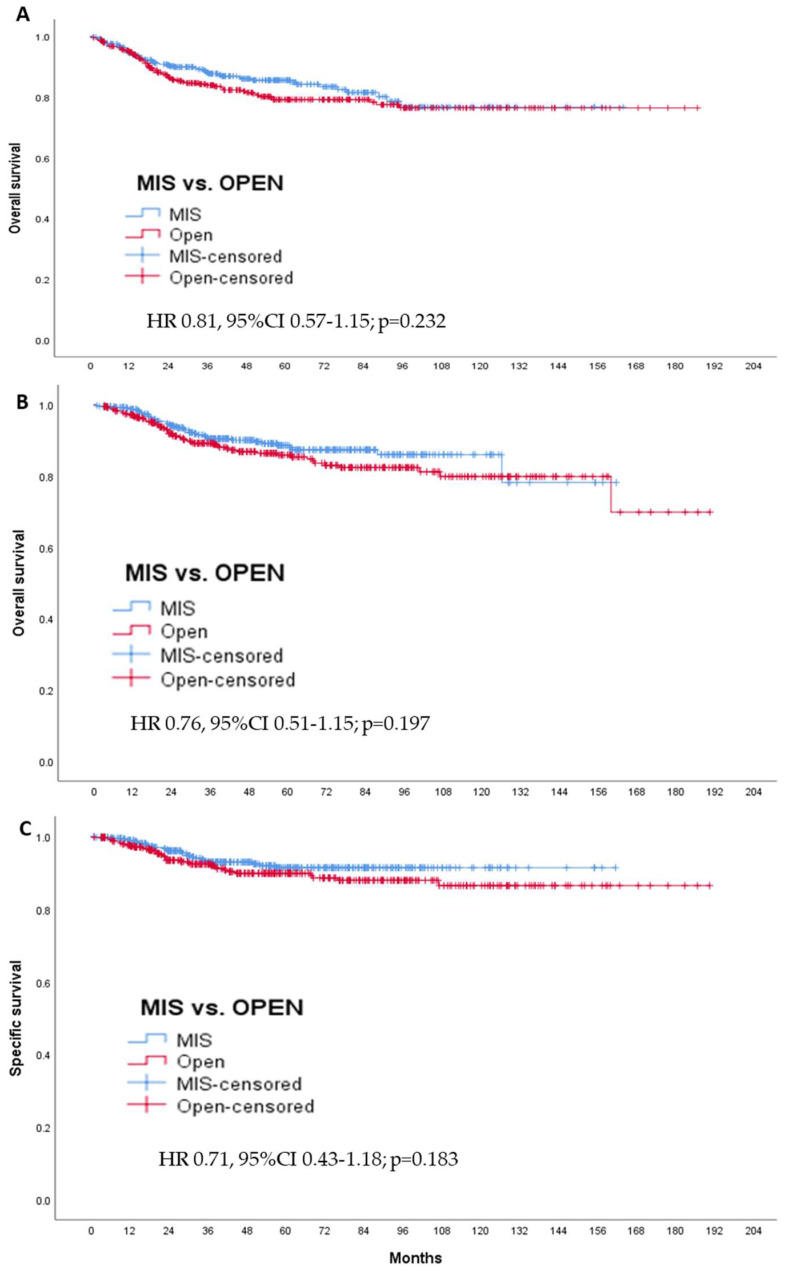
Kaplan-Meier curves for the matched surgical groups. The hazard ratio, 95% confidence interval, and corresponding *p*-values were estimated with the use of Cox’s proportional hazards model. MIS: minimally invasive surgery, indicating a laparoscopic (LPS) or robotic-assisted approach (RAL). Open: open surgery or laparotomic approach. (**A**) Disease-free survival curve, (**B**) overall survival curve, and (**C**) Specific survival curve.

The 5-year DFS percentage was 85.5% in the MIS group and 79.1% in the open surgery group (HR 0.81, 95%CI 0.57–1.15; *p* = 0.232). The 5-year OS percentage was 88.5% in the MIS group and 85.9% in the open surgery group (HR 0.76, 95%CI 0.51–1.15; *p* = 0.197). The 5-year specific survival percentage related to EC was 91.4% in the MIS group and 89.9% in the open surgery group (HR 0.71, 95%CI 0.43–1.19; *p* = 0.194) (Figure 3). Excluding FIGO stage IV from the analysis to avoid bias in the survival outcomes, similar results were obtained regarding DFS (HR 0.78, 95%CI 0.54–1.13; *p* = 0.189), OS (HR 0.74, 95%CI 0.48–1.14; *p* = 0.168), and SS (HR 0.65, 95%CI 0.38–1.12; *p* = 0.121).

When we analyzed the 399 women who underwent MIS by type of laparoscopic approach, we found that women who underwent RAL had worse histologic grades (*p* = 0.028), fewer cases that underwent para-aortic LND (*p* = 0.036), and lower rates of ESGO intermediate-risk tumors. However, there were more cases of higher-risk (*p* = 0.001) and more patients who received adjuvant chemo-radiotherapy (*p* = 0.011) (Supplementary Material Table S1).

In the survival analysis, follow-up was longer in the LPS group than in the RAL group (*p* = 0.013). The results are available in Table S2. Kaplan-Meier curves show a 5-year DFS percentage of 80.9% in the RAL group and 87.7% in the LPS group. The 5-year overall survival (OS) percentage was 85.6% in the RAL group and 89.9% in the LPS group. The 5-year survival percentage related to EC was 78.3% for RAL and 93.3% in the LPS group (Supplementary Material Figure S1). Comparing RAL and LPS groups with Cox’s analysis, the survival related to EC was higher for LPS than in the RAL group (HR: 2.23 95%CI 1.03–4.82; *p* = 0.041).

Excluding FIGO stage IV from the analysis to avoid bias in the survival outcomes, similar results were obtained with OP as a reference group regarding DFS (RAL group: HR 0.94, 95%CI 0.59–1,64; *p* = 0.942 and LPS group: HR 0.70, 95%CI 0.45–1,01; *p* = 0.095), OS (RAL group: HR 0.94, 95%CI 0.52–1.71; *p* = 0.846 and LPS group: HR 0.65, 95%CI 0.39–1.01; *p* = 0.093), and SS (RAL group: HR 1.05, 95%CI 0.53–2.08; *p* = 0.883 and LPS group: HR 0.47, 95%CI 0.24–0.94; *p* = 0.032). In SS when comparing RAL with the LPS group using Cox’s analysis, the survival related to EC was non-significantly higher for LPS than for the RAL group (HR: 2.21, 95%CI 0.95–5.10; *p* = 0.063).

## 4. Discussion

In our study, the minimally invasive approach did not seem to have any impact on the oncological outcome in patients with apparent early-stage endometrial cancer. Although a significant difference was observed in the disease-specific survival in favour of the laparoscopy group after a matched-pair analysis, no differences were shown between disease-free and overall survival.

The issue of security for minimally invasive surgery in gynaecological cancer was again raised after a recent publication in the New England Journal of Medicine [2]. The LACC trial, a phase III study published by Ramirez et al., showed a significantly higher risk of relapse and death in patients with cervical cancer from 2 to 4 cm managed with minimal invasive surgery, with a 99% 3-year overall survival after open surgery versus 93.8% after MIS (hazard ratio for death from any cause: 6.00; 95% CI, 1.77 to 20.30).

However, in EC, two randomized prospective studies have provided evidence that a minimally invasive approach is preferable in endometrial cancer management [9,10]. 

The minimally invasive approach is characterized by faster recovery, shorter hospital stays, and fewer perioperative complications, such as blood loss, thrombosis, and infections, compared to the open surgery approach. A Danish nation-wide study demonstrated that a reduction in the number of severe complications was observed despite a higher proportion of women with an older age, a high ASA score, high-risk histopathologic characteristics, and intra-abdominal adhesions offered MIS and a higher proportion of women undergoing staging lymphadenectomy [11]. Moreover, the rate of early readmission within the first 30 days after patient discharge was reduced for MIS compared to open surgery [12]. Improved quality of life in the first 6 months after MIS in all subscales related to patients dealing with cancer has been shown in randomised studies [13,14].

As far as the oncological outcome is concerned, multiple retrospective data and a pooled analysis of both prospective and retrospective studies have confirmed the superiority of MIS without an impact on survival [15,16,17,18]. 

One of the strengths of our study is that it included matched-pair analyses comparing open surgery and MIS with a homogeneous representation of non-endometrioid endometrial cancer and high-risk features by European classification in both groups. One of the largest retrospective studies on the subject concluded that women with non-endometrioid EC who underwent MIS experienced fewer complications and similar oncological outcomes compared to those who underwent open surgery [19]. A recent multicenter study by Segarra-Vidal et al. did not find difference in oncologic outcomes comparing MIS and OP among patients with high-risk endometrial cancer [20]. Another recent review on this topic published by Sacletta et al. concluded that MIS appeared to be safe in the management of high-risk EC, showing better perioperative and postoperative outcomes and comparable oncological outcomes to open surgery [21].

With the inclusion in 2018 of sentinel lymph node (SLN) mapping in the staging of high-risk endometrial cancer in the NCCN guidelines, the combination of MIS and SLN mapping could be considered for high-risk endometrial cancer when extrauterine disease is ruled out [22]. The published literature is consistent with our results, as MIS showed the same oncological safety as open surgery, even in high-risk endometrial cancer. 

Moreover, an important confounding factor in studies on surgical endometrial cancer approach is obesity, which is associated with higher operative morbidity and higher risk of conversion. In our whole series, the open surgery cohort had patients with higher BMIs, worse ASA scores, and more comorbidities compared to the MIS cohort, and the oncological outcomes showed higher survival rates among the MIS patients. However, when matched-pair modeling was performed, similar survival rates for RAL, conventional LPS, and open surgery were evidenced. Therefore, MIS in endometrial cancer seems to be oncologically safe, with quality of life improved during the first 6 months [12,13], perioperative complications not increased, and postoperative complications reduced.

Current European guidelines recommend the minimally invasive approach as the method of choice, highlighting the importance of protective maneuvers in order to avoid tumor spillage (including tumor rupture or morcellation) [23,24]. These issues were raised again after the publications the of LACC and SUCCOR trials. Along the same line, the use of a uterine manipulator, helpful in laparoscopic surgery, is another controversial topic [25]. However, current evidence is derived from retrospective studies with limited sample sizes. A recent multicenter study evaluating uterine manipulator use in early-stage endometrial cancer by MIS found a higher recurrence rate with a worse oncologic outcome in patients with uterus-confined endometrial cancer (International Federation of Gynecology and Obstetrics (FIGO) I-II), which questions the safety of the uterine manipulator in endometrial cancer [26]. 

As far as the choice of MIS technique is concerned, there has been no clear evidence for the patient benefit of robotic surgery over standard laparoscopy [27,28]. However, RAL can be a useful tool in obese patients and has shown better outcomes in this group of patients [29,30,31,32,33,34]. No studies have been published to date with adequate power to detect differences between RAL and LPS outcomes. One of the largest population-based cohort studies compared oncological outcomes according to the three surgical approaches and included 5065 patients; among them, 315 underwent RAL, 3248 underwent LPS, and 1503 underwent OP. The authors concluded that PFS was more favorable in the MIS (RAL and LPS) group than in the OP group (93.1%, 92.3%, and 87.5%, *p* < 0.001), and RAL did not seem to compromise survival outcomes when compared to LPS and OP in endometrial cancer. One limitation of this study was that the number of patients who underwent RAL was significantly lower when compared to LPS or OP groups [35]. 

One limitation of the present study was the retrospective design; however, we tried to eliminate confounding factors by the matched-pair model analyses. Another drawback was the shorter follow-up in the MIS group (especially in the RAL group) in comparison to OP, which could potentially influence the survival rates. This finding is related to the progressive incorporation of MIS in the collaborative centers and including more OP cases at the beginning. However, the median follow-up of the group had enough power to consider the results as solid. 

## 5. Conclusions

Women who underwent open surgery presented more characteristics associated with poor prognosis, and this could explain the worse survival rates found in this group compared to MIS patients. However, when comparing homogeneous groups matched by age, comorbidities, and tumor features, the survival rates between the two groups remained similar.

## Figures and Tables

**Table 1:** Patient demographics and pathology results in all whole series before matching (*n* = 1382) with EC.

Variable	MIS *N* = 684	Open *N* = 698	*p*-Value
Age (years)	65.5 ± 10.5	67.2 ± 10.7	0.003
BMI (kg/m^2^)	29.0 ± 5.8	30.2 ± 5.5	<0.001
Associated diseases ^1^	427 (62.4)	475 (68.1)	0.028
American Society of Anesthesiologists (ASA)			<0.001
I–II	480 (74.4)	344 (50.5)	
III–IV	141 (21.9)	211 (31.0)	
Unknown	24 (3.7)	126 (18.5)	
Family history of cancer			<0.001
No	431 (63.3)	520 (75.1)	
Endometrial	48 (7.0)	16 (2.3)	
Ovarian	9 (1.3)	19 (2.7)	
Breast	64 (9.4)	46 (6.6)	
Others	132 (19.0)	97 (13.3)	
Years from menopause	2.9 ± 0.4	2.9 ± 0.5	0.581
Parity	1.9 ± 1.6	2.0 ± 1.8	0.081
Histologic subtype			<0.001
Endometrioid	605 (88.5)	506 (72.5)	
Serous Papillary	44 (6.4)	88 (12.6)	
Clear cells	17 (2.5)	38 (5.4)	
Carcinosarcoma	12 (1.8)	59 (8.5)	
Undifferentiated	6 (0.9)	7 (1.0)	
Histological grade			<0.001
G1–G2	537 (78.5)	429 (61.7)	
G3	147 (21.5)	266 (38.3)	
Myometrial invasion			<0.001
<50	364 (53.2)	296 (42.4)	
≥50	320 (46.8)	402 (57.6)	
LVSI (*n* = 1330)			0.002
No	554 (82.1)	492 (75.1)	
Yes	121 (17.9)	163 (24.9)	
Postoperative stage			<0.001
Early (I–II)	621 (90.8)	578 (82.8)	
Advanced (III–IV)	63 (9.2)	120 (17.2)	
FIGO stage			<0.001
I	563 (82.3)	485 (69.5)	
II	35 (5.1)	56 (8.0)	
III	73 (10.7)	114 (16.3)	
IV	13 (1.9)	43 (6.2)	
Lymphadenectomy			0.623
No	236 (34.5)	238 (34.1)	
Pelvic only	285 (41.7)	292 (41.8)	
Pelvic and para-aortic	162 (23.7)	164 (23.5)	
Para-aortic only	1 (0.1)	4 (0.6)	
ESGO risk group			<0.001
Low	248 (36.3)	133 (19.1)	
Intermediate	166 (24.3)	170 (24.4)	
Intermediate-high	80 (11.7)	72 (10.3)	
High	177 (25.9)	280 (40.1)	
Advanced/Metastatic	14 (1.9)	43 (6.1)	
Adjuvant therapies			<0.001
No	274 (40.1)	218 (31.2)	
Radiotherapy	332 (48.5)	331 (47.4)	
Chemotherapy	10 (1.5)	32 (4.6)	
Radio-chemotherapy	68 (9.9)	117 (16.8)	
Length of follow-up (months)	57.3 ± 34.5	63.5 ± 46.4	0.012

Data are given as mean ± standard deviation and frequencies (percentages). BMI: body mass index. LVSI: lymphovascular space invasion. ^1^ Includes tobacco smoking, diabetes, cardiovascular diseases, thromboembolic disease, chronic pneumopathies, and liver diseases.

**Table 2:** Patient demographics and pathology results in MIS subanalysis (*n* = 684) with EC.

Variable	Robotic *N* = 233	LPS *N* = 451	*p*-Value
Age (years)	65.7 ± 10.3	65.5 ± 10.6	0.800
BMI (kg/m^2^)	29.2 ± 5.9	28.9 ± 5.8	0.404
Associated diseases ^1^	145 (62.2)	282 (62.5)	0.940
American Society of Anesthesiologists (ASA)			0.016
I–II	165 (72.2)	315 (75.4)	
III–IV	59 (26.0)	82 (19.6)	
Unknown	3 (1.3)	21 (5.0)	
Years from menopause	2.9 ± 0.3	2.9 ± 0.2	0.762
Parity	1.7 ± 0.1	1.5 ± 0.1	0.063
Histologic subtype			0.373
Endometrioid	204 (87.6)	401 (88.9)	
Serous Papillary	16 (6.9)	28 (6.2)	
Clear cells	5 (2.1)	12 (2.7)	
Carcinosarcoma	7 (3.0)	5 (1.1)	
Undifferentiated	1 (0.4)	5 (1.1)	
Histological grade			0.051
G1–G2	173 (74.2)	364 (80.7)	
G3	60 (25.8)	87 (19.3)	
Myometrial invasion			0.052
<50	136 (58.4)	228 (50.6)	
≥50	97 (41.6)	223 (49.4)	
LVSI (*n* = 1330)			0.039
No	201 (86.3)	353 (79.9)	
Yes	32 (13.7)	89 (20.1)	
Postoperative stage			0.479
Early (I–II)	209 (89.7)	412 (91.4)	
Advanced (III–IV)	24 (10.3)	39 (8.6)	
FIGO stage			0.421
I	185 (79.4)	378 (83.8)	
II	12 (5.2)	23 (5.1)	
III	30 (12.9)	43 (9.5)	
IV	6 (2.6)	7 (1.6)	
Lymphadenectomy			<0.001
No	74 (31.8)	162 (35.9)	
Pelvic only	120 (51.5)	165 (36.6)	
Pelvic and para-aortic	38 (16.3)	124 (27.5)	
Para-aortic only	1 (0.4)	0 (0)	
ESGO risk group			<0.001
Low	103 (44.2)	145 (32.2)	
Intermediate	41 (17.6)	125 (27.7)	
Intermediate-high	15 (6.4)	65 (14.4)	
High	68 (29.2)	109 (24.2)	
Advanced/Metastatic	6 (2.6)	7 (1.6)	
Adjuvant therapies			0.012
No	100 (42.9)	174 (38.6)	
Radiotherapy	96 (41.2)	236 (52.3)	
Chemotherapy	5 (2.10)	5 (1.1)	
Radio-chemotherapy	32 (13.7)	36 (8.0)	
Lenth of follow up	50.8 ± 30.2	60.6 ± 36.0	0.013

Data are given as mean ± standard deviation and frequencies (percentages). BMI: body mass index. LVSI: lymphovascular space invasion. LPS: laparoscopic approach. ^1^ Includes: tobacco smoking, diabetes, cardiovascular diseases, thromboembolic disease, chronic pneumopathies, and liver diseases.

**Table 3:** Patient demographics and pathology results in matched-pair analysis (*n* = 798).

Variable	MIS *N* = 399	Open *N* = 399	*p*-Value
Age (years)	66.4 ± 10.4	66.0 ±10.8	0.861
BMI (kg/m^2^)	30.0 ± 5.8	30.4 ± 5.5	0.318
Associated diseases ^1^	270 (67.7)	270 (67.7)	>0.999
American Society of Anesthesiologists (ASA)			>0.999
I–II	268 (67.2)	268 (67.2)	
III–IV	102 (25.6)	102 (25.6)	
Unknown	29 (7.2)	29 (7.2)	
Years from menopause	2.9 ± 0.4	2.8 ± 0.5	0.063
Parity	2.1 ± 1.9	2.1 ± 1.9	0.996
Histologic subtype			>0.999
Endometrioid	345 (86.5)	345 (86.5)	
Serous Papillary	31 (7.8)	31 (7.8)	
Clear cells	12 (3.0)	12 (3.0)	
Carcinosarcoma	9 (2.3)	9 (2.3)	
Undifferentiated	2 (0.5)	2 (0.5)	
Histological grade			>0.999
G1–G2	295 (73.9)	295 (73.9)	
G3	104 (26.1)	104 (26.1)	
Myometrial invasion			>0.999
<50	189 (47.4)	189 (47.4)	
≥50	210 (52.6)	210 (52.6)	
LVSI (*n* = 765)			0.349
No	320 (81.6)	314 (83.7)	
Yes	72 (18.4)	59 (15.7)	
Postoperative Stage			>0.999
Early (I–II)	346 (86.7)	346 (86.7)	
Advanced (III–IV)	53 (13.3)	53 (13.3)	
FIGO stage			0.623
I	324 (81.2)	318 (79.7)	
II	22 (5.5)	29 (7.3)	
III	45 (11.3)	41 (10.0)	
IV	8 (2.0)	12 (3.0)	
Lymphadenectomy			0.175
No	118 (29.6)	130 (32.6)	
Pelvic only	169 (42.4)	184 (46.1)	
Pelvic and para-aortic	111 (27.8)	84 (21.1)	
Para-aortic only	1 (0.3)	1 (0.3)	
ESGO risk group			0.837
Low	112 (28.1)	112 (28.1)	
Intermediate	115 (28.8)	121 (30.3)	
Intermediate-high	46 (11.5)	40 (10.0)	
High	118 (29.6)	114 (28.6)	
Advanced/Metastatic	8 (2.0)	12 (3.0)	
Adjuvant therapies			0.423
No	142 (35.6)	144 (36.1)	
Radiotherapy	204 (51.1)	192 (48.1)	
Chemotherapy	7 (1.8)	14 (3.5)	
Radio-chemotherapy	46 (11.5)	49 (12.3)	
Length of follow-up	57.7 ± 34.5	63.5 ± 46.4	0.012

Data are given as mean ± standard deviation and frequencies (percentages). BMI: body mass index. LVSI: lymphovascular space invasion. LPS: laparoscopic approach. ^1^ Includes: tobacco smoking, diabetes, cardiovascular diseases, thromboembolic disease, chronic pneumopathies, and liver diseases.

## Data Availability

The data presented in this study are available on request from the corresponding author. The data are not publicly available due to their containing information that could compromise the privacy of patients.

## Supplementary Materials

The following supporting information can be downloaded at: https://www.mdpi.com/article/10.3390/cancers14041081/s1, Table S1: Cox’s Multivariant analysis to identify the independent prognostic variables associated to disease free survival and overall survival, Table S2: Patient demographics and pathology results in MIS matched-pair analysis. Figure S1: Kaplan–Meier curves for the matched surgical group.

## References

[1] Ferlay J., Colombet M., Soerjomataran I., Mathers C., Parkin D.M., Piñeros M., Znaor A., Bray F. (2018). Estimating the global cancer incidence and mortality in 2018: GLOBOCAN sources and methods. Int. J. Cancer.

[2] Ramirez P.T., Frumovitz M., Pareja R., López A., Vieira M., Ribeiro M., Buda A., Yan X., Shuzhong Y., Chetty N. (2018). Minimally invasive versus abdominal radical hysterectomy for cervical cancer. N. Engl. J. Med..

[3] Melamed A., Margul D.J., Chen L., Keating N.L., Del Carmen L.G., Yang J., Seagle B.L., Alexander A., Barber E.L., Rice L.W. (2018). Survival after minimally invasive radical hysterectomy for early-stage cervical cancer. N. Engl. J. Med..

[4] Walker J.L., Piedmonte M.R., Spirtos N.M., Eisenkop S.M., Schlaerth J.B., Mannel R.S., Spiegel G., Barakat R., Pearl M.L., Sharma S.K. (2009). Laparoscopy compared with laparotomy for comprehensive surgical staging of uterine cancer: Gynecologic Oncology Group Study LAP2. J. Clin. Oncol..

[5] Jørgensen S.L., Mogensen O., Chunsen S., Korsholm M., Lund K., Jensen P. (2019). Survival after a nationwide introduction of robotic surgery in women with early-stage endometrial cancer: A population-based prospective cohort study. Eur. J. Cancer.

[6] Cuschieri S. (2019). The STROBE guidelines. Saudi J. Anaesth..

[7] Oncoguía SEGO (ES) (2010). Cancer de Endometrio 2010. Guías de Práctica Clínica en Cancer Ginecologico y.

[8] Colombo N., Creutzberg C., Amant F., Bosse T., Martín A.G., Ledermann J., Marth C., Nout A.R., Querleu D., Mirza M.R. (2016). ESMO-ESGO-ESTRO Consensus Conference on Endometrial Cancer: Diagnosis, Treatment and Follow-up. Int. J. Gynecol. Cancer.

[9] Janda M., Gebski V., Davies L.C., Forder P., Brand A., Hogg R., Jobling T.W., Land R., Manolitsas R., Nascimento M. (2017). Effect of Total Laparoscopic Hysterectomy vs Total Abdominal Hysterectomy on Disease-Free Survival Among Women With Stage I Endometrial Cancer: A Randomized Clinical Trial. JAMA.

[10] Walker J.L., Piedmonte M.R., Spirtos N.M., Eisenkop S.M., Schlaerth J.B., Mannel R., Barakat R., Pearl M., Sudarshan K.S. (2012). Recurrence and survival after random assignment to laparoscopy versus laparotomy for comprehensive surgical staging of uterine cancer: Gynecologic Oncology Group LAP2 Study. J. Clin. Oncol..

[11] Jørgensen S.L., Mogensen O., Wu C., Lund K., Iachina M., Korsholm M., Jensen P.T. (2019). Nationwide Introduction of Minimally Invasive Robotic Surgery for Early-Stage Endometrial Cancer and Its Association With Severe Complications. JAMA Surg..

[12] Beck T.L., Schiff M.A., Goff B.A., Urban R.R. (2018). Robotic, laparoscopic or open hysterectomy: Surgical outcomes by approach in endometrial cancer. J. Minim. Invasive Gynecol..

[13] Janda M., Gebski V., Brand A., Hogg R., Jobling T.W., Land R., Manolitsas T., McCartney A., Nascimento M., Neesham D. (2010). Quality of life after total laparoscopic hysterectomy versus total abdominal hysterectomy. Lancet Oncol..

[14] Zullo F., Palomba S., Russo T., Falbo A., Constantino M., Tolino A., Zupi E., Tagliaferri P., Venuta S. (2005). A prospective randomized comparison between laparoscopic and laparotomic approaches in women with early-stage endometrial cancer: A focus on the quality of life. Am. J. Obstet. Gynecol..

[15] Park D.A., Lee D.H., Kim S.W., Lee S.H. (2016). Comparative safety and effectiveness of robot-assisted laparoscopic hysterectomy versus conventional laparoscopy and laparotomy for endometrial cancer: A systematic review and meta-analysis. Eur. J. Surg. Oncol..

[16] Kyrgiou M., Swart A.M., Qian W., Warwick J. (2015). A Comparison of Outcomes Following Laparoscopic and Open Hysterectomy With or Without Lymphadenectomy for Presumed Early-Stage Endometrial Cancer: Results From the Medical Research Council ASTEC Trial. Int. J. Gynecol. Cancer.

[17] Asher R., Obermair A., Janda M., Gebski V. (2018). Disease-Free and Survival Outcomes for Total Laparoscopic Hysterectomy Compared With Total Abdominal Hysterectomy in Early-Stage Endometrial Carcinoma: A Meta-analysis. Int. J. Gynecol. Cancer.

[18] Coronado P.J., Herráiz M.A., Magrina J.F., Fasero M., Vidart J.A. (2012). Comparison of perioperative outcomes and cost of robotic-assisted laparoscopy, laparoscopy and laparotomy for endometrial cáncer. Eur. J. Obstet. Gynecol. Reprod Biol..

[19] Monterossi G., Ghezzi F., Vizza E., Zannoni G.F., Uccela S., Corrado G., Restaino S., Quagliozzi L., Casarin J., Dinoi G. (2017). Minimally invasive approach in type II endometrial cáncer: Is it wise and safe?. J. Minim. Invasive Gynecol..

[20] Segarra-Vidal B., Dinoi G., Zorrilla-Vaca A., Mariani A., Student V., Garcia N.A., Abella A.L., Ramirez P.T. (2021). Minimally Invasive Compared With Open Hysterectomy in High-Risk Endometrial Cancer. Obstet. Gynecol..

[21] Scaletta G., Dinoi G., Capozzi V., Cianci S., Pelligra S., Ergasti R., Fagotti A., Scambia G., Fanfani F. (2020). Comparison of minimally invasive surgery with laparotomic approach in the treatment of high-risk endometrial cancer: A systematic review. Eur. J. Surg. Oncol..

[22] Koh W.J., Abu-Rustum N., Bean S., Bradley K., Campos S., Cho K.R., Chon H.S., Chu C., Cohn D., Crispens M.A. (2018). Uterine Neoplasms, Version 1.2018, NCCN Clinical Practice Guidelines in Oncology. J. Natl. Compr. Can. Netw..

[23] Concin N., Matias-Guiu X., Vergote I., Cibula D., Mirza M.R., Marnitz S., Ledermann J., Bosse T., Chargari C., Fagotti A. (2021). ESGO/ESTRO/ESP guidelines for the management of patients with endometrial carcinoma. Int. J. Gynecol. Cancer.

[24] Concin N., Planchamp F., Abu-Rustum N., Ataseven B., Cibula D., Fagotti A., Fotopoulou C., Knapp P., Marth C., Morice P. (2021). European Society of Gynaecological Oncology quality indicators for the surgical treatment of endometrial carcinoma. Int. J. Gynecol. Cancer.

[25] Padilla-Iserte P., Quintana R., Marina T., Lago V., Matute L., Domingo S. (2021). Uterine manipulator in endometrial cancer: A video is worth a thousand words. Int. J. Gynecol. Cancer.

[26] Padilla-Iserte P., Lago V., Tauste C., Díaz-Feijoo B., Gil-Moreno A., Oliver R., Coronado P., Salamanca M.B.M., Pantoja-Garrido M., Marcos-Sanmartin J. (2021). Impact of uterine manipulator on oncological outcome in endometrial cancer surgery. Am. J. Obstet. Gynecol..

[27] Ran L., Jin J., Xu Y., Youquan B., Song F. (2014). Comparison of robotic surgery with laparoscopy and laparotomy for treatment of endometrial cancer: A meta-analysis. PLoS ONE.

[28] Gala R.B., Margulies R., Steinberg A., Murphy M., Lukban J., Jeppson P., Aschkenazi S., Olivera C., South M., Lowenstein L. (2014). Systematic review of robotic surgery in gynecology: Robotic techniques compared with laparoscopy and laparotomy. J. Minim. Invasive Gynecol..

[29] Gracia M., García-Santos J., Ramírez M., Bellón M., Herráiz M.A., Coronado P. (2020). Value of robotic surgery in endometrial cancer by body mass index. Int. J. Gynaecol. Obstet.

[30] Subramaniam A., Kim K.H., Bryant S.A., Zhang B., Sikes C., Kimball K.J., Kilgore L.C., Huh W.K., Straughn J.M., Alvarez R.D. (2011). A cohort study evaluating robotic versus laparotomy surgical outcomes of obese women with endometrial carcinoma. Gynecol. Oncol..

[31] Seamon L.G., Bryant S.A., Rheaume P.S., Kimball K.J., Huh W.K., Fowler J.M., Phillips G.S., Cohn D.E. (2009). Comprehensive surgical staging for endometrial cancer in obese patients: Comparing robotics and laparotomy. Obstet Gynecol..

[32] Rebeles S.A., Muntz H.G., Wieneke-Broghammer C., Vason E., McGonigle K.F. (2009). Robot-assisted total laparoscopic hysterectomy in obese and morbidly obese women. J. Robot. Surg..

[33] Lau S., Buzaglo K., Vaknin Z., Brin S., Kaufer R., Drummond N., Gourdji I., Aubin S., Rosberger Z., Gotlieb W.H. (2011). Relationship between body mass index and robotic surgery outcomes of women diagnosed with endometrial cancer. Int. J. Gynecol. Cancer.

[34] Bernardini M.Q., Gien L.T., Tipping H., Murphy J., Rosen B.P. (2012). Surgical outcome of robotic surgery in morbidly obese patient with endometrial cancer compared to laparotomy. Int. J. Gynecol. Cancer.

[35] Eoh K.J., Nam E., Kim S.W., Shin M., Kim S., Kim J., Kim Y. (2021). Nationwide Comparison of Surgical and Oncologic Outcomes in Endometrial Cancer Patients Undergoing Robotic, Laparoscopic, and Open Surgery: A Population-Based Cohort Study. Cancer Res. Treat..

