# Supplementary material for: Survival Analysis in Endometrial Carcinomas by Type of Surgical Approach: A Matched-Pair Study
Source: Cancers (Basel). 2022 Feb 21;14(4):1081. doi: 10.3390/cancers14041081 (PMC8870025; doi:10.3390/cancers14041081)
Supplement: Supplementary file 1 [file cancers-14-01081-s001.zip › cancers-1574693-supplementary.pdf]

Supplementary Material: Survival Analysis in Endometrial Carcinomas by Type of Surgical Approach: A Matched-Pair Study

Pluvio J. Coronado, Agnieszka Rychlik, Laura Baquedano, Virginia García-Pineda, Maria A. Martínez-Maestre, Denis Querleu, Ignacio Zapardiel

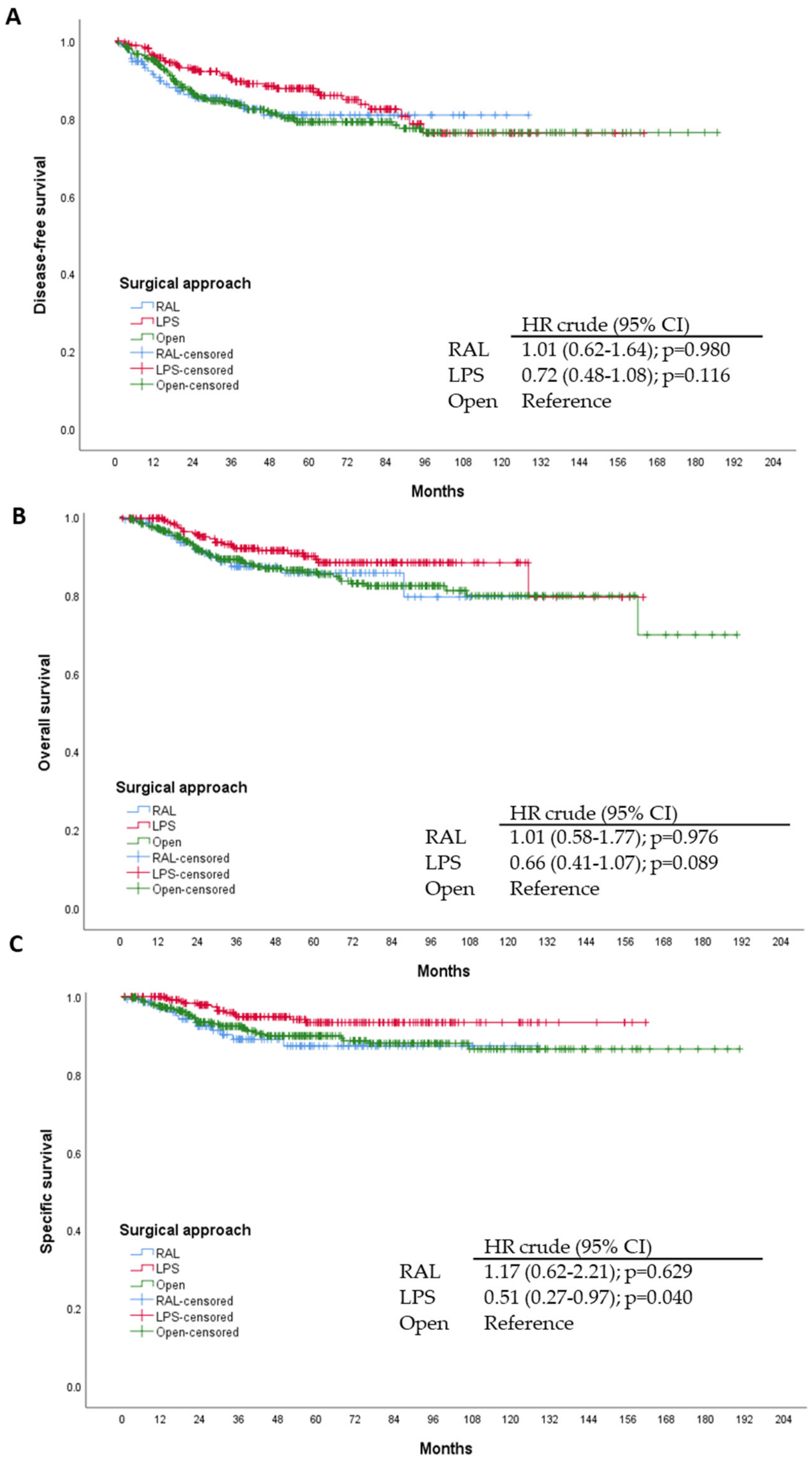

Figure S1. Kaplan–Meier curves for the matched surgical groups. The hazard ratio, 95% confidence interval, and corresponding p value were estimated with the use of Cox proportional-hazards models. MIS: Minimally invasive surgery, indicates laparoscopic (LPS) or robot-assisted approach (RAL). Open: open surgery or laparotomic approach. A: disease-free survival curve. B: overall survival curve. C: specific survival curve.

Table S1. Cox's Multivariant analysis to identify the independent prognostic variables associated to disease free survival and overall survival.

	**Disease Free Survival**								
		**B**	**SE**	**Wald**	**df**	**Sig.**	**Exp(B)**	**CI 95% Exp(B)**	
								**Inferior**	**Superior**
Step 1	Age (years)	0.028	0.007	15.013	1	0.000	1.028	1.014	1.042
	BMI >30 kg/m^2	−0.072	0.138	.274	1	0.601	0.930	.709	1.220
	ASA >III	0.262	0.102	6.581	1	0.010	1.299	1.064	1.587
	Associated diseases [1]	−0.354	0.144	6.028	1	0.014	0.702	0.529	0.931
	Histological subtype (endometriod)	−0.408	0.181	5.071	1	0.024	0.665	0.466	0.948
	Histological grade 3	0.802	0.180	19.812	1	0.000	2.230	1.566	3.174
	Deep myometrial invasion	0.461	0.139	10.936	1	0.001	1.586	1.207	2.084
	Advanced FIGO stage	1.080	0.160	45.571	1	0.000	2.944	2.152	4.029
Step 2	Age (years)	0.028	0.007	14.966	1	0.000	1.028	1.014	1.042
	ASA >III	0.256	0.102	6.332	1	0.012	1.291	1.058	1.576
	Associated diseases [1]	−0.370	0.141	6.839	1	0.009	0.691	0.524	0.912
	Histological subtype (endometriod)	−0.419	0.180	5.404	1	0.020	0.658	0.462	0.936
	Histological grade 3	0.805	0.180	20.068	1	0.000	2.236	1.573	3.180
	Deep myometrial invasion	0.464	0.139	11.108	1	0.001	1.590	1.211	2.089
	Advanced FIGO stage	1.078	0.160	45.474	1	0.000	2.939	2.149	4.021
	**Overall Survival**								
		**B**	**SE**	**Wald**	**df**	**Sig.**	**Exp(B)**	**CI 95% Exp(B)**	
								**Inferior**	**Superior**
Strep 1	Age (years)	0.045	0.008	33.955	1	0.000	1.046	1.030	1.062
	BMI >30 kg/m^2	0.023	0.142	0.027	1	0.870	1.023	0.775	1.351
	ASA >III	0.609	0.101	36.403	1	0.000	1.839	1.509	2.242
	Associated diseases [1]	−0.317	0.158	4.019	1	0.045	0.728	0.534	0.993
	Histological subtype (endometriod)	−0.430	0.186	5.327	1	0.021	0.651	0.452	0.937
	Histological grade 3	1.071	0.188	32.398	1	0.000	2.919	2.019	4.222
	Deep myometrial invasion	0.659	0.155	18.135	1	0.000	1.933	1.427	2.618
	Advanced FIGO stage	0.846	0.170	24.651	1	0.000	2.330	1.669	3.254
Step 2	Age (years)	0.045	0.008	33.948	1	0.000	1.046	1.030	1.062
	ASA >III	0.611	0.101	36.941	1	0.000	1.842	1.513	2.243
	Associated diseases [1]	−0.312	0.155	4.046	1	0.044	0.732	0.540	0.992
	Histological subtype (endometriod)	−0.427	0.185	5.305	1	0.021	0.653	0.454	0.938
	Histological grade 3	1.071	0.188	32.343	1	0.000	2.918	2.018	4.221
	Deep myometrial invasion	0.658	0.155	18.098	1	0.000	1.931	1.426	2.614
	Advanced FIGO stage	0.846	0.170	24.652	1	0.000	2.330	1.669	3.254

BMI: Body Mass index. ASA: American Society of Anesthesiology. 1Includes tobacco smoking, diabetes, cardiovascular diseases, thromboembolic disease, chronic pneumopathies and liver diseases.

Table S2. Patient demographics and pathology results in MIS matched-pair analysis (n = 399).

	**Robotic *N*= 134**	**LPS *N*= 265**	***p* Value**
Age (years)	66.5 ± 10.0	66.5 ± 10.7	0.971
BMI (kg/m2)	30.1 ± 5.8	29.8 ± 5.9	0.688
Associated diseases1	87 (64.9)	183 (69.1)	0.405
American Society of Anesthesiologists (ASA)			0.057

I–II	91 (67.9)	177 (66.8)	
III–IV	40 (29.9)	62 (23.4)	
Unknown	3 (2.2)	26 (9.8)	
Years from menopause	2.9 ± 0.5	2.9 ± 0.5	0.946
Parity	1.7 ± 0.1	1.5 ± 0.1	0.078
Histologic subtype			0.248
Endometrioid	112 (83.6)	233 (87.9)	
Serous Papillary	14 (10.4)	17 (6.4)	
Clear cells	3 (2.2)	9 (3.4)	
Carcinosarcoma	5 (3.7)	4 (1.5)	
Undifferentiated	0 (0)	2 (0)	
Histological grade			0.028
G1–G2	90 (67.2)	205 (77.4)	
G3	44 (32.8)	60 (22.6)	
Myometrial invasion			0.070
< 50	72 (53.7)	117 (44.2)	
≥ 50	62 (46.3)	148 (55.8)	
LVSI (*n* = 765)			0.205
No	114 (85.1)	206 (77.8)	
Yes	20 (14.9)	52 (20.2)	
Stage at diagnosis			0.825
Early (I–II)	122 (91.0)	243 (91.7)	
Advanced (III–IV)	12 (9.0)	22 (8.3)	
FIGO stage			0.465
I	103 (76.9)	221 (83.4)	
II	9 (6.7)	13 (4.9)	
III	19 (14.2)	26 (9.8)	
IV	3 (2.2)	5 (1.9)	
Lymphadenectomy			0.036
No	40 (29.9)	78 (29.4)	
Pelvic only	66 (49.3)	103 (38.9)	
Pelvic and paraaortic	27 (20.1)	84 (31.7)	
Paraaortic only	1 (0.7)	0 (0)	
ESGO risk group			0.001
Low	47 (35.1)	65 (24.5)	
Intermediate	26 (19.4)	89 (33.6)	
Intermediate-high	8 (6.0)	38 (14.3)	
High	50 (37.3)	68 (25.7)	
Advanced/Metastatic	3 (2.2)	5 (1.9)	
Adjuvant therapies			0.011
No	51 (38.1)	91 (34.3)	
Radiotherapy	56 (41.8)	148 (55.8)	
Chemotherapy	4 (3.0)	3 (1.1)	
Radio-chemotherapy	23 (17.2)	23 (8.7)	
Follow-up in alive	48.7 ± 30.1	58.9 ± 34.6	0.003

Data are given as mean ± standard deviation and frequencies (percentages). BMI: Body Mass index. LVSI: lymphovascular space invasion. LPS: laparoscopic approach. 1 Includes: tobacco smoking, diabetes, cardiovascular diseases, thromboembolic disease, chronic pneumopathies and liver diseases.
